# Development of a cell adhesion-based prognostic model for multiple myeloma: Insights into chemotherapy response and potential reversal of adhesion effects

**DOI:** 10.32604/or.2023.043647

**Published:** 2024-03-20

**Authors:** QIAN HU, MENGYAO WANG, JINJIN WANG, YALI TAO, TING NIU

**Affiliations:** Department of Hematology, West China Hospital, Sichuan University, Chengdu, 610041, China

**Keywords:** Cell adhesion, Bioinformatics, Prognosis, Multiple myeloma, CAM-DR

## Abstract

Multiple myeloma (MM) is a hematologic malignancy notorious for its high relapse rate and development of drug resistance, in which cell adhesion-mediated drug resistance plays a critical role. This study integrated four RNA sequencing datasets (CoMMpass, GSE136337, GSE9782, and GSE2658) and focused on analyzing 1706 adhesion-related genes. Rigorous univariate Cox regression analysis identified 18 key prognosis-related genes, including KIF14, TROAP, FLNA, MSN, LGALS1, PECAM1, and ALCAM, which demonstrated the strongest associations with poor overall survival (OS) in MM patients. To comprehensively evaluate the impact of cell adhesion on MM prognosis, an adhesion-related risk score (ARRS) model was constructed using Lasso Cox regression analysis. The ARRS model emerged as an independent prognostic factor for predicting OS. Furthermore, our findings revealed that a heightened cell adhesion effect correlated with tumor resistance to DNA-damaging drugs, protein kinase inhibitors, and drugs targeting the PI3K/Akt/mTOR signaling pathway. Nevertheless, we identified promising drug candidates, such as tirofiban, pirenzepine, erlotinib, and bosutinib, which exhibit potential in reversing this resistance. *In vitro*, experiments employing NCIH929, RPMI8226, and AMO1 cell lines confirmed that MM cell lines with high ARRS exhibited poor sensitivity to the aforementioned candidate drugs. By employing siRNA-mediated knockdown of the key ARRS model gene KIF14, we observed suppressed proliferation of NCIH929 cells, along with decreased adhesion to BMSCs and fibronectin. This study presents compelling evidence establishing cell adhesion as a significant prognostic factor in MM. Additionally, potential molecular mechanisms underlying adhesion-related resistance are proposed, along with viable strategies to overcome such resistance. These findings provide a solid scientific foundation for facilitating clinically stratified treatment of MM.

## Introduction

Multiple myeloma (MM) is the second most common hematological malignancy and is characterized by the excessive proliferation of plasma cells in the bone marrow [1]. To date, MM remains incurable in most patients, with drug resistance driving relapse. Cell adhesion-mediated drug resistance (CAM-DR) is a major clinical problem that prevents successful treatment of MM [2].

Cell adhesion encompasses interactions between cells and the extracellular matrix (ECM), as well as between cells themselves. In MM, this adhesion effect predominantly occurs between MM cells and bone marrow stromal cells, as well as ECM proteins within the bone marrow microenvironment [3]. Previous research underscores the pivotal role of cell adhesion in various facets of MM, including disease onset, the occurrence of minimal residual disease (MRD), tumor dormancy, extramedullary dissemination (to the lungs, liver, pleura, and peritoneal fluid), drug resistance, and the modulation of the bone marrow immune microenvironment [4–6]. Additionally, studies suggest that MM may induce heightened osteoclast activity through adhesion and interaction, consequently driving more osteolytic events and exacerbating myeloma-associated bone disease.

Cell adhesion is integral throughout the entirety of MM development, emphasizing its paramount importance in disease progression. In recent years, an increasing number of cell adhesion-related molecules and pathways have been found to exhibit abnormal expression in MM pathogenesis. Consequently, targeted inhibitors or modulators developed in this context have garnered significant attention. Integrins are heterodimeric membrane glycoprotein receptors that facilitate the interaction between the extracellular matrix and cell-cell adhesion molecules. Specifically, α4β1 (VLA-4) and α4β7 have been established to exert a substantial influence on MM cell adhesion, migration, homing, invasion, and drug resistance. Notably, studies by Vandyke et al. and Groen et al. have demonstrated upregulated expression of N-cadherin on MM cells in approximately 50% of newly diagnosed MM patients [7]. The adhesion pathway involving SDF-1 (CXCL12) and its receptor CXCR4, expressed on MM cells, has been extensively investigated in MM homing. The CXCR4 inhibitor AMD3100 and the anti-CXCR4 antibody MAB171 impede the homing of MM cells to bone marrow niches, making them potential agents for MM treatment [8]. CD38, a receptor involved in regulating cell adhesion, migration, and signal transduction, plays a pivotal role in the bone marrow microenvironment of MM. Daratumumab is the first therapeutic monoclonal anti-CD38 antibody approved by the Federal Drug Agency (FDA) for the treatment of MM both as a single agent and in combination with lenalidomide or bortezomib [9]. Additionally, immunomodulatory drugs (IMiDs) such as lenalidomide, bortezomib, and natalizumab are prominent examples of drugs that interfere with cell adhesion and have been developed for MM treatment [4,10]. Thus, the exploration of the role and interplay of cell adhesion molecules in multiple myeloma provides valuable insights into potential therapeutic targets for overcoming clinical drug resistance.

While numerous studies have highlighted the significance of individual cell adhesion in the progression of MM, a comprehensive investigation in this area is lacking. In the current study, we conducted a comprehensive analysis of 1706 cell adhesion-related genes (ARGs) to assess their prognostic value in MM using multiple datasets. Additionally, we developed an adhesion-related risk (ARR) model to predict patient outcomes. Moreover, we explored drug sensitivity among different ARR groups and predicted candidate drugs to overcome CAM-DR. Furthermore, we validated our bioinformatic findings *in vitro* using MM cell lines. Our study not only revealed novel molecular mechanisms underlying cell adhesion but also presented opportunities for the development of personalized therapeutic strategies in MM.

## Materials and Methods

### Public data acquisition and preprocessing

The RNA-seq transcripts per million (TPM) data of 775 newly diagnosed MM (NDMM) patients from the multiple myeloma research foundation (MMRF) CoMMpass cohort were downloaded from the GDC data portal (https://portal.gdc.cancer.gov/projects/MMRF-COMMPASS/). Two external independent datasets, GSE2658 containing 410 NDMM patients and GSE136337 containing 422 NDMM patients, were downloaded from the GEO database and quantitated by Affymetrix Human Genome U133 Plus 2.0 Array. Another independent dataset of GSE9782 containing 264 MM patients was downloaded from the GEO database and quantitated by Affymetrix Human Genome U133A Array. The summarized clinical and laboratory profiles of patients within the CoMMpass, GSE136337, and GSE9782 datasets are presented in Suppl. Table S1.

### Adhesion-related gene collection

A total of 1706 adhesion-related genes were collated from Gene Ontology Biological Process (GOBP) and Kyoto Encyclopedia of Genes and Genomes (KEGG).

### Univariate and multivariate Cox analysis

Univariate Cox analysis was conducted to screen and validate significant prognostic adhesion-related genes. Forest plots reporting the *p* value, HR, and 95% CI were drawn using R language.

Risk scores were analyzed using both univariate and multivariate Cox proportional hazards models to assess whether they were independent prognostic factors. We considered factors with hazard ratios (HRs) > 1 to be prognostic risk factors and those with HRs < 1 to be prognostic protective factors.

### Construction of an adhesion-related risk model

The 18 common adhesion-related genes correlated with overall survival in four datasets were identified by an online tool generating a Venn diagram (http://bioinformatics.psb.ugent.be/webtools/Venn). Then, these 18 candidate genes were used to construct a multigene signature for predicting overall survival (OS) prognosis. The CoMMpass dataset was designated as the training set to construct the prognostic model, while the other three GEO datasets were designated as the test sets to verify the result of the training set. Finally, 12 adhesion-related genes were identified to construct the risk model by least absolute shrinkage and selection operator (LASSO) Cox regression analysis using the R package “glmnet” [11]. Using the linear combination of gene expression weighted regression coefficients, we obtained the adhesion-related risk score formula: risk score = (exp MSN*0.155) − (exp PECAM1*0.096) + (exp HTR2C*0.040) + (exp GTPBP4*0.186) + (exp LGALS1*0.055) − (exp ALCAM*0.053) − (exp MERTK*0.029) + (exp KIF14*0.326) + (exp TENM1*0.042) − (exp JUN*0.025) + (exp FLNA*0.111) + (exp TROAP*0.211). Patients were divided into low-and high-risk groups based on their median risk score. Kaplan‒Meier (KM) survival curves and time-dependent receiver operating characteristic (ROC) analysis were performed to evaluate the prognostic value of the adhesion-related risk score using the “survival”, “survminer” and “timeROC” packages. Risk curves and scatter plots were generated to show the risk score and vital status of individuals.

### Establishment of the nomogram

Following collation with clinical information, the clinicopathologic features of 612 patients in the CoMMpass dataset and 416 MM patients in the GSE136337 dataset were employed to construct the prognostic nomogram. Then, the nomograms and calibration curves were plotted with the “rms” package.

### Interaction network

To further explore the interactome of 18 prognostic adhesion-related genes, the “igraph” and “reshape2” R packages were used to calculate Pearson correlation coefficients and build the gene coexpression network. In addition, the online tool STRING (http://string-db.org/) was applied to construct the protein‒protein interaction network of those 18 genes at the protein level.

### Functional enrichment analysis

Gene set enrichment analysis (GSEA) and gene set variation analysis (GSVA) were performed to obtain the biological significance correlated with the adhesion-related risk score. Briefly, GSEA was performed on the entire group of transcriptomes by using GSEA software (http://software.broadinstitute.org/gsea/). Gene sets with *p* values < 0.05 were considered statistically significant and were then plotted by the “ggplot2” R package. GSVA was implemented to estimate each gene set variation in every patient by using the “GSVA” and “GSEABase” R packages. The differences in biological pathways between high-and low-risk patients were determined by the “limma” R package, and an adjusted *p* value < 0.05 was considered statistically significant.

FunRich software was used to identify the primary molecular function of the genes included in the risk model.

### Prediction of chemotherapy sensitivity

Based on the Genomics of Drug Sensitivity in Cancer (GDSC) v2 (https://www.cancerrxgene.org/) data portal, the drug half inhibitory concentration (IC50) values of each sample were evaluated using the calcPhenotype algorithm of the “oncoPredict” R package in CoMMpass and GSE136337. The Wilcoxon test was performed to describe the differences in drug sensitivity between diverse risk groups.

Genes differentially expressed in the high-risk group compared to the low-risk group were identified using the “limma” R package. The 32 common upregulated genes and 78 common downregulated genes identified in both the CoMMpass and GSE136337 datasets were uploaded into the Connectivity Map (cMAP) (https://clue.io/query). Millions of drugs were identified with similar or opposite gene-regulation pattern inputs. The connectivity score of each drug ranged from −1 to +1, and those drugs with values closer to −1 had a reversible gene-drug relationship, suggesting that they could overcome the adhesion effect in the high-risk group.

### Cell culture and chemicals

RPMI8226, NCIH929 and AMO1 cells were cultured in RPMI 1640 medium with 10% fetal bovine serum (FBS, Gibco) and 1% penicillin/streptomycin (HyClone).

AZD5363 and pictilisib were purchased from Bidepharm (Shanghai, China). Uprosertib, dabrafenib and foretinib were purchased from Aladdin (Shanghai, China). Nutlin-3a and selumetinib were purchased from MedChemExpress (Shanghai, China).

### Cell proliferation and apoptosis assessment

For the cell proliferation assay and determination of IC50, we employed a Cell Counting Kit-8 (CCK-8, Yeasen, Shanghai, China) following the manufacturer’s protocol.

Apoptosis analysis was conducted using an Annexin V-FITC/PI Apoptosis Detection Kit (Yeasen, Shanghai, China).

### Cell transfection

KIF14 siRNAs were custom-synthesized by GenePharma (Shanghai, China) and transiently transfected into cells using Lipofectamine 2000 transfection reagent (Invitrogen, CA, USA) following the manufacturer’s instructions. The specific sequences for KIF14 siRNAs were as follows: #489 (AUAUCAAGAAUAUCACCGCTT), #3021 (AUAAUACUCACUGUCCCACTT), and #4950 (UUUAAGAAUUCUGGAGCACTT).

### Real-time qPCR and Western blot analysis

Total RNA was extracted from cells using the HiPure Universal RNA Mini Kit (Magen, China). The RNA concentration was quantified using a Nanodrop 2000 spectrophotometer (Thermo Fisher Scientific, USA). Subsequently, the RNA was reverse transcribed into cDNA using the TransScript All-in-One First-Strand cDNA Synthesis SuperMix for qPCR (One-Step gDNA Removal) Kit. The resulting cDNA served as the template for qPCR, which was carried out using Hieff qPCR SYBR Green Master Mix (YEASEN, Shanghai, China) on a real-time fluorescence quantitative PCR instrument (LongGene, Hangzhou, China). The expression levels of target genes were normalized to GAPDH. The primer sequences can be found in Suppl. Table S2.

Western blot analysis was conducted as previously described [12]. The primary antibody against KIF14 was procured from Bethyl Laboratories (A300-233A-T).

### Cell adhesion assay

For the adhesion assays, we utilized soluble fibronectin (FN) or HS-5 cells. Initially, Petri plates were coated with HS-5 cells or 40 μg/mL FN (MedChemExpress, Shanghai, China) in 1 mL of culture medium or phosphate-buffered saline (PBS), respectively [13]. These plates were then incubated overnight in a 37°C incubator. Simultaneously, NCIH929 cells were transfected with either siNC or siKIF14#4950 using Lipofectamine 2000 overnight. Subsequently, NCIH929 cells were labeled with 5 μM calcein-AM (Beyotime, Shanghai, China) for 0.5 h, strictly following the manufacturer’s instructions. Next, the PBS-washed NCIH929 cells (2 × 10^5^ cells/mL) were allowed to adhere to a preestablished monolayer of HS-5 or FN. After 6 h of adhesion, the plates underwent thorough washing three times with PBS. Finally, images were captured using a fluorescence microscope (Nikon, Tokyo, Japan).

### Statistical analysis

Data are expressed as the mean ± standard deviation (SD). All experiments were independently performed in triplicate. Statistical significance was assessed using either two-way analysis of variance (ANOVA) or Student’s *t-*test, as appropriate. GraphPad Prism 8.0 software was employed for data analysis and visualization. A *p* value < 0.05 was considered statistically significant.

## Results

### Establishment of adhesion-related prognostic model

**Figure 1:**
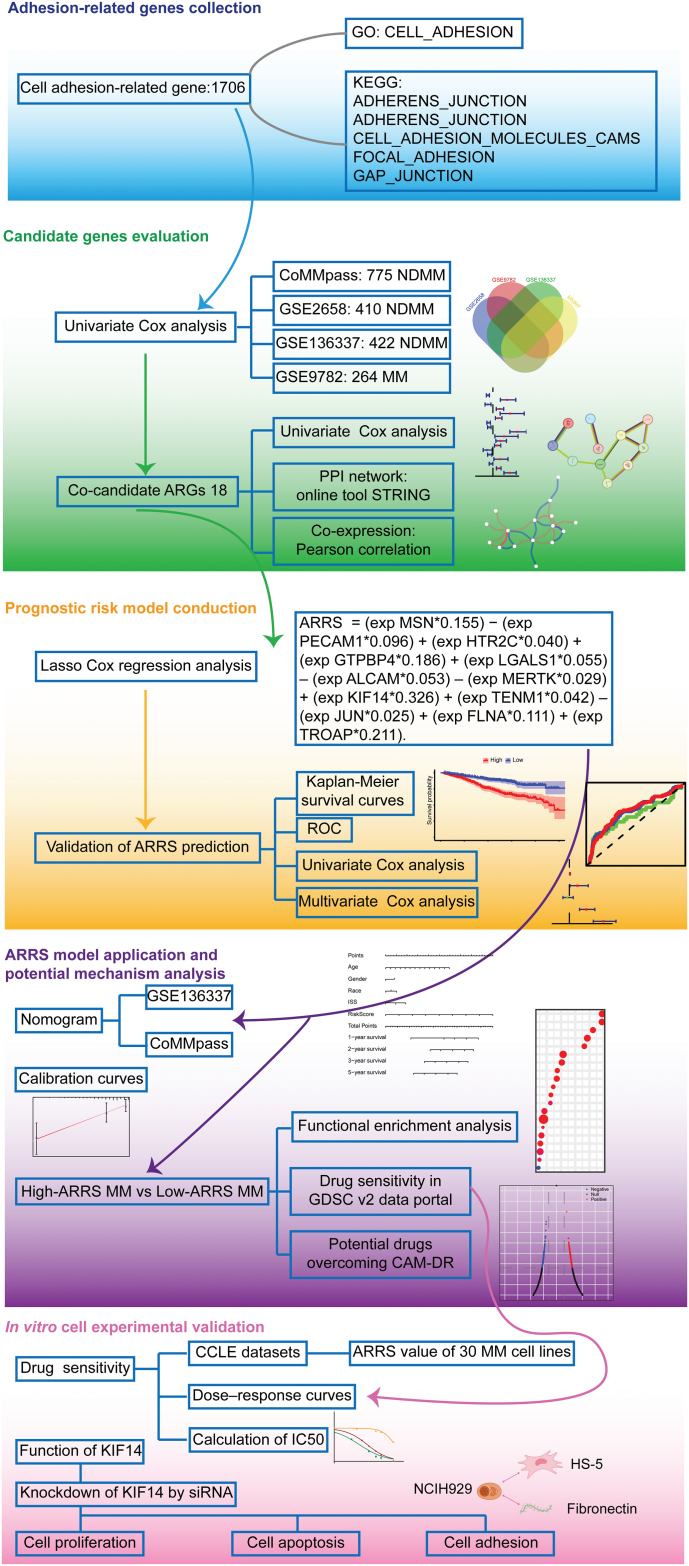
Flow chart of bioinformatics analysis for adhesion-related genes associated with MM prognosis. Initially, we collected cell adhesion-related genes from the GO and KEGG databases, resulting in 1706 genes. Through independent prognostic analysis, we identified 18 key genes associated with prognosis across four datasets. To understand their interactions and relationship with prognosis, we performed independent prognostic analysis, PPI network analysis, and coexpression network analysis. Subsequently, we constructed a prognostic model for cell adhesion-related genes using Lasso Cox regression analysis, ensuring its reliability and accuracy through various validation methods. Based on the adhesion-related risk score (ARRS) model, we developed a nomogram tool for disease prognosis prediction and confirmed its accuracy. Following this, we stratified patients into high-and low-ARRS groups using the ARRS values. We conducted molecular pathway enrichment analysis and drug sensitivity analysis for both groups while also predicting potential drugs with the ability to reverse adhesion effects. Finally, we employed MM cell lines to validate the predicted drug sensitivities and employed siRNA to knockdown the key model gene KIF14 to investigate its role in tumor progression and adhesion.

The flow chart of the study is presented in Fig. 1.

**Figure 2:**
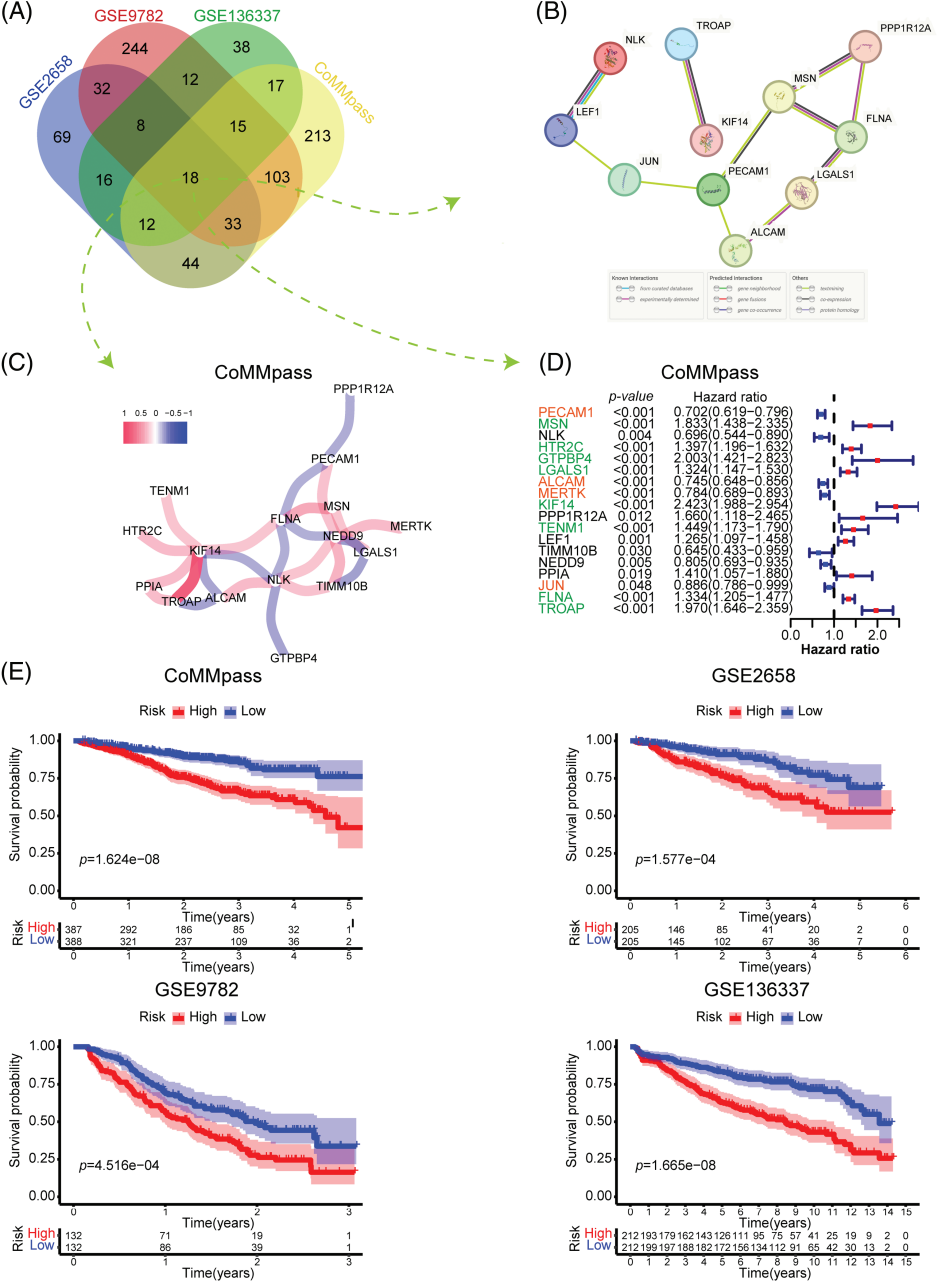
Identification and construction of the adhesion-related gene prognostic model. (A) Venn diagram showing the number of prognostic adhesion-related genes overlapping in the CoMMpass, GSE136337, GSE9782 and GSE2658 datasets (plotted using http://bioinformatics.psb.ugent.be/webtools/Venn/). (B and C) Protein‒protein interaction (PPI) network (B) and coexpression network (C) of 18 adhesion-related genes that were significantly associated with prognosis in all four datasets. (D) Forest plot showing the univariate Cox regression results of 18 adhesion-related genes in the CoMMpass dataset. (E) Kaplan‒Meier (KM) survival curves comparing overall survival between low-and high-risk patients in the CoMMpass, GSE136337, GSE9782 and GSE2658 datasets.

To investigate the most significant ARGs associated with OS in MM patients, we selected 4 MM datasets with relatively large sample sizes for prognosis analysis. Univariate Cox regression analysis was applied to identify ARGs with significant prognostic value (*p* < 0.05). As depicted in the Venn diagram, 18 ARGs correlated with OS overlapped in all 4 datasets (Fig. 2A). To further explore the interaction of these 18 prognostic ARGs, we constructed a PPI network using the STRING tool (Fig. 2B). A total of 11 ARGs were identified in this interaction network. In addition, we performed Pearson correlation analysis of 18 intersecting ARGs in the CoMMpass dataset and constructed a coexpression network with a coefficient threshold of 0.3 (Fig. 2C). To identify the prognostic value of each candidate ARG, we reperformed univariate regression analysis of those genes in 4 MM datasets (Fig. 2D and Suppl. Fig. S1). Notably, KIF14, a gene encoding a member of the kinesin-3 superfamily of microtubule motor proteins, interacted extensively with other genes and was strongly associated with poor prognosis (HR = 2.423, *p* < 0.001). TROAP, a gene encoding trophinin-associated protein, is needed for microtubular cytoskeleton regulation, centrosome integrity, and spindle assembly during mitosis. TROAP can facilitate cell adhesion during embryo implantation [14]. There was a significant interaction between KIF14 and TROAP, whether it was determined through experimental evidence or coexpression analysis (Figs. 2B and 2C). Although both KIF14 and TROAP have been identified as oncogenes in various solid tumors [15,16], their involvement in MM is still uncertain.

FLNA, a gene encoding filamin A (FLNa), participates in the formation of the cytoskeleton and regulates cell adhesion and migration. By anchoring a variety of proteins in the cytoskeleton, FLNa acts broadly on signal transduction, proliferation, differentiation and chemoresistance [17]. In fact, FLNa plays dual roles in cancer. Full-length FLNa located in the cytoplasm promotes cell proliferation and migration, while cleaved FLNa located in the nucleus inhibits cell proliferation by negatively regulating the synthesis of rRNA [18]. Our data indicate that FLNA significantly correlated with poor OS in MM (HR = 1.334, *p* < 0.001), which has not been reported previously. MSN encodes moesin, which acts as a structural linker between the plasma membrane and the actin filaments in the cell, regulating fundamental cellular processes such as cell morphology, adhesion, and motility. The effect of moesin on hematologic malignancy has been poorly studied. However, recent research suggests that moesin could be a potential therapeutic target to address bortezomib resistance in MM [19]. LGALS1, a gene encoding galectin-1 (Gal-1), serves as a potential therapeutic target for cancer treatment [20]. As reported, Gal-1 functions in diverse processes of tumor progression, including proliferation, migration and adhesion, immune responses, inflammation, intercellular and cell–matrix interactions and carcinogenesis [21]. A similar trend was found in this study: LGALS1 was negatively correlated with MM prognosis (HR = 1.324, *p* < 0.001). A previous review indicated that platelet endothelial cell adhesion molecule-1 (PECAM-1/CD31) is involved in inhibiting apoptosis and thus contributes to resistance to chemotherapeutic treatment in various types of tumors, including hematologic malignancies [22]. ALCAM, also known as CD166, is a transmembrane glycoprotein weighing 105 kDa and belongs to the immunoglobulin superfamily. Many previous studies have shown that ALCAM is linked to the development of several types of solid cancer and acute myeloid leukemia. Thus, ALCAM has been identified as a potential therapeutic target through monoclonal antibody or specific chimeric antigen receptor T-cell (CAR-T cell) cancer treatment [23,24]. However, our results showed a positive effect of PECAM1 (HR = 0.702, *p* < 0.001) and ALCAM (HR = 0.745, *p* < 0.001) on the OS of MM. Further research is required to validate the precise function of these 18 candidate ARGs in MM.

To investigate the overall prognostic correlation of ARGs, we performed Lasso Cox regression analysis of 18 prognosis-related ARGs and identified 12 genes to construct the adhesion-related prognostic gene signature (the colored genes in Fig. 2D). We calculated the adhesion-related risk score (ARRS) of each patient based on the coefficient of each gene in the risk model. Using the median ARRS of each dataset, we divided MM patients into high- or low-risk groups (Suppl. Fig. S2A). As shown in Suppl. Fig. S2B, the patients with a higher ARRS tended to die more likely during the follow-up. In addition, Kaplan‒Meier analysis confirmed that patients with a high ARRS had significantly shorter overall survival, which was consistent in all four datasets (Fig. 2E). According to the findings presented above, the ARRS is a significant adverse prognostic factor in MM.

### ARRS is an independent predictor of overall survival in MM patients

**Figure 3:**
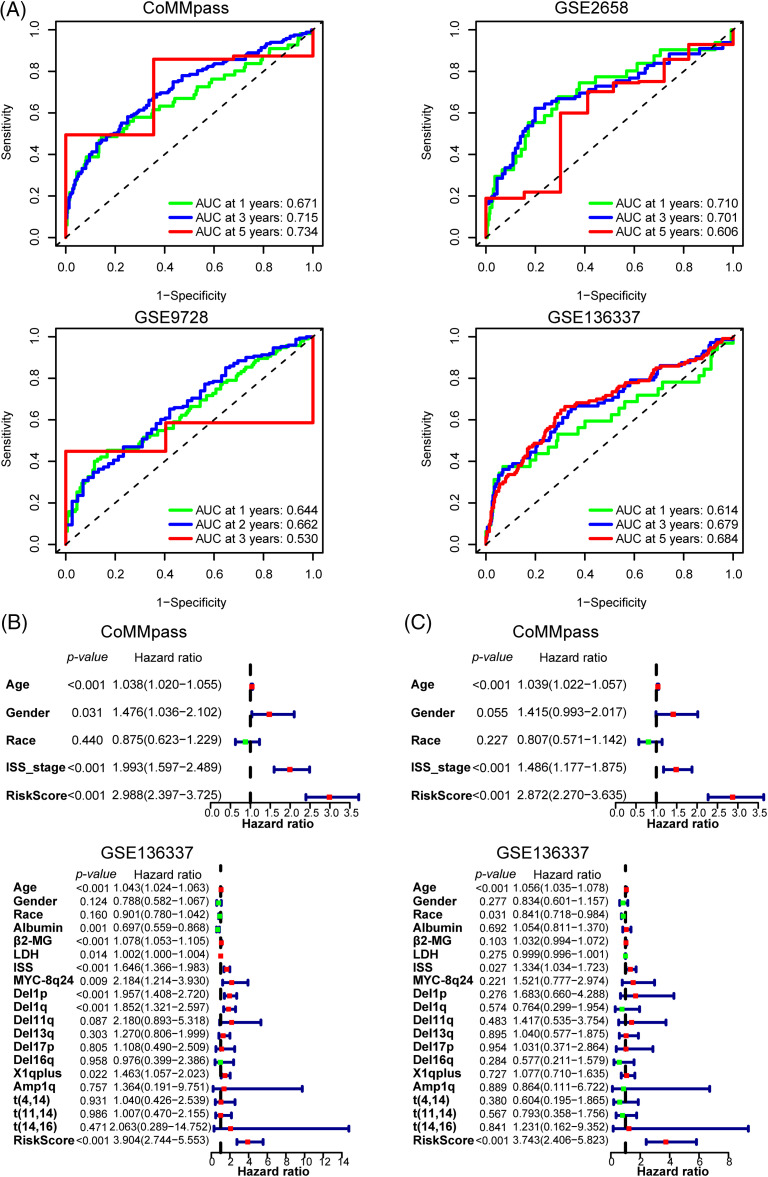
Validation of the predictive value of the 12 adhesion-related gene-based risk model. (A) Time-dependent ROC curves for the adhesion-related risk prediction model in the CoMMpass, GSE136337, GSE9782 and GSE2658 datasets. (B and C) Forest plots showing the univariate (B) and multivariate Cox regression (C) analyses of risk score and clinical features in relation to overall survival in the CoMMpass and GSE136337 datasets.

To assess the predictive efficacy of the risk model for prognosis, we utilized ROC analysis, as well as univariate and multivariate Cox regression analyses with ARRS to assess its prognostic value. In the CoMMpass dataset, the areas under the ROC curve (AUCs) after 1, 3, and 5 years were 0.671, 0.715, and 0.734, respectively (Fig. 3A). In both the GSE2658 and GSE136337 datasets, the AUC consistently exceeded 0.6, regardless of the cutoff years. However, in the GSE9728 dataset, the AUC was above 0.6 for the first two years but decreased to 0.53 after 3 years due to a smaller sample size with longer follow-up periods (Fig. 3A). These findings suggest that the ARRS has significant predictive ability for prognosis in MM. Both single-factor independent prognostic analysis and multivariate independent prognostic analysis showed that ARRS, age and international staging system (ISS) stage were independent prognostic factors in the CoMMpass datasets (*p* < 0.001). Similarly, ARRS, age and ISS stage were highly statistically significant independent predictors of prognosis in GSE136337 (*p* < 0.001) (Figs. 3B and 3C).

Additionally, in univariate analysis for GSE136337, albumin, β2-microglobulin (β2-MG), and lactate dehydrogenase (LDH) levels, in addition to MYC-8q24, del(1p), del(1q), and X1q plus chromosomal abnormalities, were identified as independent prognostic factors for the OS of MM patients. However, in a multivariate analysis, none of these factors remained significant. More interestingly, among all the clinical factors considered, ARRS had the highest hazard ratio values (HR = 2.872 to 3.904), indicating that it is the strongest predictor of a worse clinical outcome in MM. In general, the ARRS can serve as an independent and powerful prognostic predictor, which is even more robust than classical predictors for predicting survival in MM.

### Construction of a prognostic nomogram incorporating the ARRS and clinical parameters

To develop a useful prognostic tool that integrates the ARRS with other potential prognostic factors, we constructed nomograms using the CoMMpass and GSE136337 datasets. Nomograms are valuable for quantitatively assessing patient prognosis, providing clinicians with a reliable basis for clinical decision-making. As shown in Suppl. Fig. S3A, ARRS contributed the most to prognosis, even more than ISS stage and other clinical features in the CoMMpass nomogram. Additionally, calibration curves for 1-, 3-, 5-, and 10-year OS showed high consistency between the nomogram predictions and actual observations (Suppl. Fig. S3B). Similarly, in the GSE136337 dataset, ARRS accounted for the highest proportion of total points in the nomogram (Suppl. Fig. S3A). The accuracy of the nomogram was also validated by calibration curves (Suppl. Fig. S3B). As more clinical features were collected in the GSE136337 dataset, older age, higher β2-MG levels, higher LDH levels, and later ISS stages were associated with worse survival rates. Additionally, the occurrence of chromosomal abnormalities, including MYC-8q24, del(1p), del(11q), del(13q), del(17p), X1qplus, and t(14,16), was associated with poor prognosis. Overall, we successfully constructed nomograms for predicting survival probabilities at various time points in MM patients.

### Investigation of risk-related KEGG pathways by GSVA and GSEA

In our study, we constructed a risk model based on ARGs that calculated the risk score by multiplying the log2-transformed normalized gene expression with a negative or a positive coefficient. Only 12 genes were taken into account for the calculation. However, ARRS values may not consistently correspond to the extent of the cells’ adhesion capability. To determine this, we used Funrich to assess the primary molecular function of the 12 genes included in the model. The results demonstrated that cell adhesion was the most prominent function of these genes, which confirmed our initial hypothesis (Suppl. Fig. S4A). Next, we employed GSEA to identify signaling pathways related to cell adhesion in MM. As shown in Suppl. Fig. S4B, the cell adhesion and GAP junction pathways (GJs) were significantly enriched in the high-ARRS group compared to the low-ARRS group. These results suggest that the high-ARRS group exhibited higher levels of cell adhesion, particularly in GJ activation. GJs, which are composed of connexins, enable the direct transfer of ions and small molecules (Ca^2+^, glutamate, ATP, or NAD^+^) between cells. Connexins are widely expressed in human cells, functioning as electrical communicators in excitable cells, such as neurons. In addition, connexins mediate the exchange of small metabolites and second messengers to regulate cell proliferation and differentiation and the maintenance of tissue homeostasis [25,26]. In addition to intercellular channels, the intercellular domains of connexins can also interact with other proteins, including components of the cytoskeleton and cell signaling pathways [27]. Accumulating investigations have shown that GJs are a double-edged sword in cancer therapy. On the one hand, GJs can promote the cytotoxicity of various chemotherapeutic drugs and induce the widespread bystander effect of radiotherapy. On the other hand, GJs can provide some substances to sustain cancer dormancy and may adversely affect the efficacy of anticancer drugs and cause cellular resistance [28]. However, the exact role of GJs in MM is not yet clear. In our study, we were the first to elucidate that high-ARRS populations had a higher biological effect of cell adhesion, resulting in worse prognosis compared to low-risk populations in MM.

**Figure 4:**
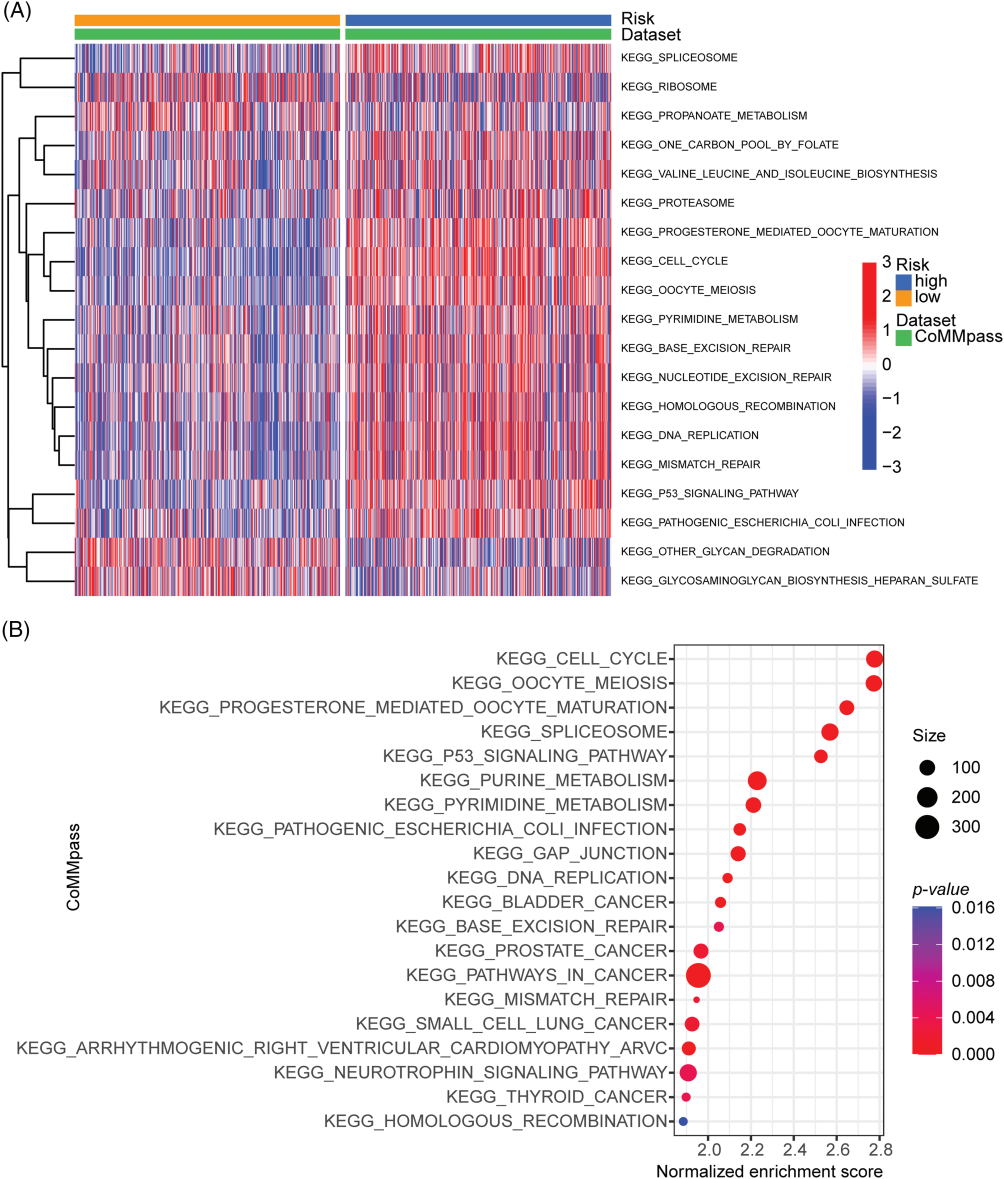
GSVA and GSEA enrichment analysis of the activation status of biological pathways in distinct adhesion-related risk groups. (A) Heatmap visualizing the enrichment scores of gene set variation analysis (GSVA) in the CoMMpass cohort. (B) Bubble plot showing the significantly enriched KEGG pathways in the high-risk group compared to the low-risk group identified by gene set variation analysis (GSVA) in the CoMMpass cohort.

To investigate the critical mechanisms or pathways that contributed to the prognostic differences between high- and low-ARRS MM patients, we applied GSVA and GSEA enrichment analysis. Both GSVA and GSEA were performed for each subgroup of genes in various signaling pathways. However, GSEA was performed between the high- and low-ARRS groups, and GSVA calculated the gene set signatures in each patient first, which required further statistical analysis to define the differences between groups. Consistently identified by GSVA and GSEA, the cell cycle, spliceosome, DNA replication, base excision repair, mismatch repair, homologous recombination and P53 signaling pathways were significantly enriched in high-ARRS MM patients in the CoMMpass dataset (Fig. 4). The results obtained from the GSE136337 dataset contributed to the same conclusion (Suppl. Fig. S5). The dysregulation of the cell cycle is a prevalent hallmark of cancer, characterized by uncontrolled cell proliferation. Remarkably, the expression levels of cell cycle transcripts have demonstrated substantial potential in predicting drug sensitivity [29]. Furthermore, base excision repair, mismatch repair and homologous recombination are critical components of DNA damage response (DDR) pathways, which protect cells against exogenous or endogenous DNA damage. Cytogenetic abnormalities are the main hallmark of MM. Most of the drugs currently used to treat MM have direct genotoxic activity (i.e., melphalan, doxorubicin, cyclophosphamide) or interfere with the DNA repair machinery (protease inhibitors or IMiDs). Once the DDR pathways are overactivated, the efficiency of cytotoxic therapy is considerably reduced [30,31]. In addition, the upregulation of the P53 signaling pathway may contribute to excessive activation of DDR [32]. Therefore, we hypothesized that DDR-mediated chemoresistance may be part of the reason for the shorter survival time in the high-ARRS group than in the low-ARRS group.

### Evaluation of the therapeutic response in different adhesion-related risk groups

**Figure 5:**
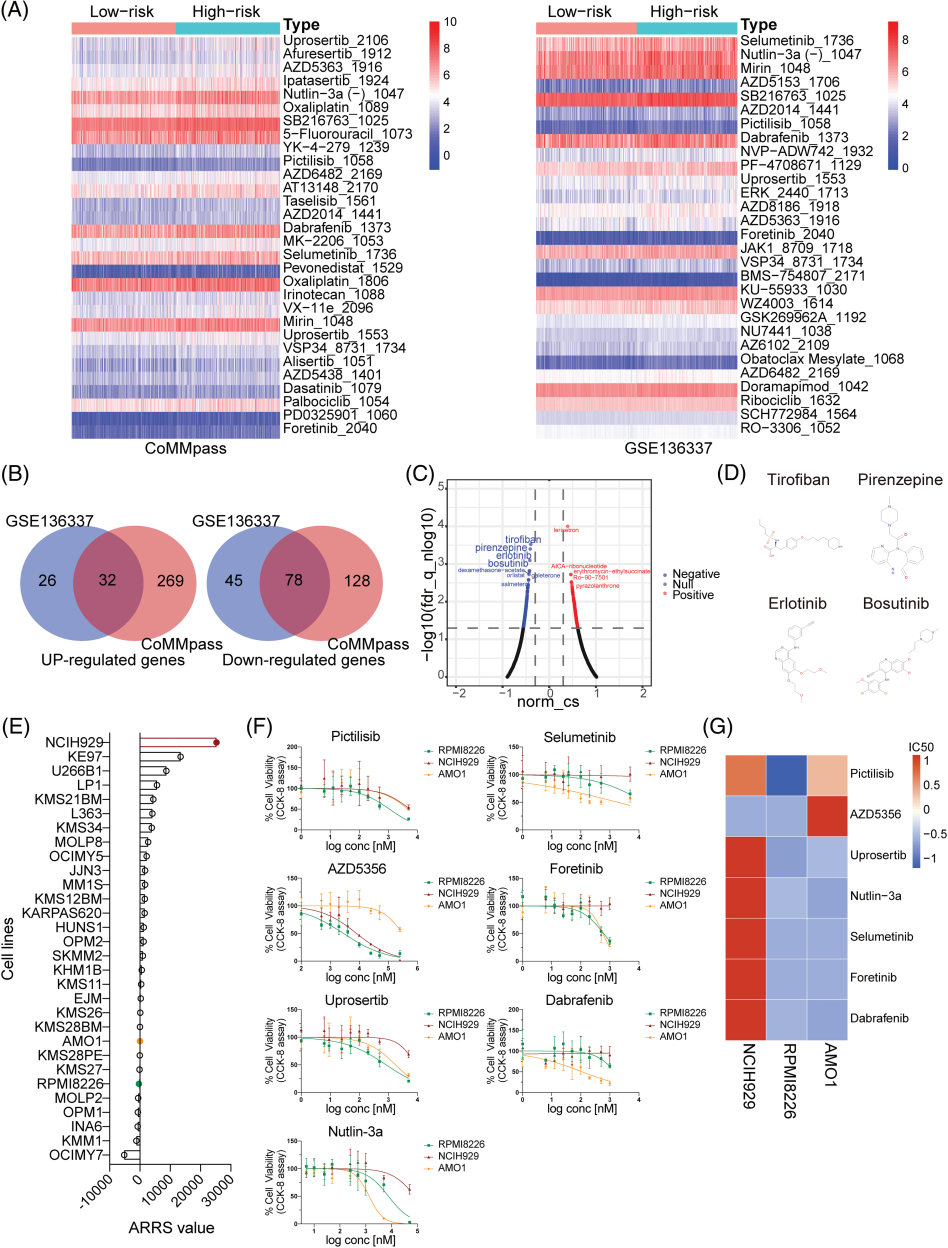
Analysis, prediction, and validation of drug sensitivity in different ARRS groups. (A) Heatmaps visualizing the drug efficiency (IC50) in various risk patients evaluated by the “oncoPredict” R package based on the genomics of drug sensitivity in cancer (GDSC) v2. The color scale indicates the log2-transformed IC50. IC50, half-maximal inhibitory concentration. (B) Venn diagrams showing significantly upregulated and downregulated genes in high-risk patients compared to low-risk patients in the CoMMpass and GSE136337 datasets. (C) Volcano plot showing drugs with negative or positive gene-regulation patterns predicted using the connectivity map (cMAP) by inputting 32 upregulated and 78 downregulated genes identified in (B). (D) Chemical structures of the first four negative drugs in C. (E) Histogram depicting the distribution of ARRS values across 30 MM cell lines utilizing expression data sourced from the Cancer Cell Line Encyclopedia (CCLE) datasets. (F) Assessment of cell viability in NCIH929, RPMI8226, and AMO1 cell lines in response to candidate drugs associated with CAM-DR. (G) Heatmaps illustrating the drug efficacy (IC50) in diverse adhesion-related risk MM cell lines.

Building upon previous prognostic and pathway enrichment analyses, our study demonstrated that the high-ARRS group exhibited inferior survival outcomes compared to the low-ARRS group, which could be attributed to the hyperactivation of DNA damage response pathways and other survival pathways. To further assess the sensitivity of MM patients with diverse ARRS to various therapeutic compounds, the oncoPredict package was applied to calculate the IC50 of agents in the GDSC v2 database. In the CoMMpass dataset, 87 of 156 total predicted drugs had higher IC50 values in the high-ARRS group than in the low-ARRS group, and the rest of the predicted drugs remained constant in diverse groups. The top 30 drugs with the most significant IC50 differences are shown in the heatmap. In the GSE136337 dataset, 29 of 156 total predicted drugs expressed higher IC50 values in the high-ARRS group than in the low-ARRS group. After analyzing the common drugs that showed significant differences in drug response between the CoMMpass and GSE136337 datasets, we identified six types of PI3K/Akt/mTOR signaling pathway inhibitors (AZD2014, AZD5363, AZD6482, pictilisib, uprosertib, and VSP34) that exhibited higher resistance in the high-ARRS group than in the low-ARRS group (Fig. 5A). Additionally, we found that oxaliplatin, a DNA alkylating agent causing DNA damage, was less effective in the high-ARRS group than in the low-ARRS group. In addition, many targeted agents were found to be resistant in the high-ARRS group, including dabrafenib (a selective BRAF inhibitor), foretinib (a multikinase inhibitor), mirin (an MRN-ATM pathway inhibitor), Nutlin-3a (−) (an MDM2 antagonist and p53 activator), SB216763 (a GSK-3 inhibitor) and selumetinib (a MEK 1/2 inhibitor). BRAF mutations are detected in ~7%–15% of all cancers, including hairy cell leukemia (79%–100%), melanomas (40%–70%), papillary thyroid cancers (45%), ovarian cancers (35%), and multiple myeloma (4%). Although the FDA granted accelerated approval to dabrafenib for the treatment of tumors harboring a BRAFV600 mutation, dabrafenib has shown limited efficacy in hematological malignancies in clinical trials, unlike in other solid tumors [33,34]. Our study revealed for the first time that high cellular adhesive effects may mediate adverse reactions to dabrafenib. In a phase II clinical trial conducted in 2016, selumetinib administered in relapsed and refractory MM only achieved a response rate of 5.6%; therefore, it is not recommended as a monotherapy for the clinical treatment of MM [35]. Our study suggests that treatment failure of selumetinib may be related to cell adhesion. Overall, the above results suggest that MM patients in the high-ARRS group may be resistant to protein kinase inhibitors, DNA damaging drugs, and PI3K/Akt/mTOR signaling pathway targeted drugs compared to low-ARRS MM.

To provide novel strategies for drug tolerance and poor prognosis in high-ARRS MM patients, we applied cMAP database to screen candidate drugs with opposite or similar expression patterns to high-ARRS MM. First, we summarized the common upregulated and downregulated genes in the high ARRS group compared to the low ARRS group in the CoMMpass and GSE136337 datasets (Fig. 5B). Using those 32 common upregulated genes and 78 downregulated genes as input, we identified some potential drugs with negative scores for high-ARRS MM therapy (Fig. 5C). The structures of the top four predicted drugs are shown in Fig. 5D. Interestingly, tirofiban is an antiplatelet drug that inhibits platelet aggregation by inhibiting the surface membrane of platelet αIIb/β3 integrin. As reported, integrin αIIb/β3 participates in tumor cell proliferation and metastasis, and whether targeting αIIb/β3 benefits MM patients remains unclear [36,37]. Pirenzepine is a cholinergic antagonist. The content of cholinergic system compartments is altered in many cancers and correlates with patient survival in some cancers [38]. There is substantial epidemiological evidence to support the notion that occupational exposure to cholinergic compounds (namely, pesticides) is linked to an elevated risk of developing MM [39]. Whether pirenzepine can prevent the pathogenesis of MM caused by pesticides and be applied in high-ARRS MM therapy deserves further investigation. Both erlotinib and bosutinib are orally administered tyrosine kinase inhibitors (TKIs). The FDA has approved erlotinib for treating non-small cell lung cancer and pancreatic cancer and bosutinib for first-line treatment of chronic myeloid leukemia [40–42]. In addition, erlotinib has been demonstrated to be beneficial for patients suffering from MDS or AML [43]. Despite the lack of direct clinical evidence, our study has identified potential drugs for the treatment of high-ARRS MM patients, which warrants further investigation in the design of clinical trials.

### Assessment of drug sensitivity disparities in MM cell lines with diverse ARRS values

To further investigate the relationship between ARRS and drug sensitivity *in vitro*, we initially obtained the gene expression profiles of 30 multiple myeloma (MM) cell lines from the Cancer Cell Line Encyclopedia (CCLE) database. Subsequently, we calculated the ARRS scores for each cell line using the ARRS formula. As depicted in Fig. 5E, NCIH929 exhibited the highest ARRS score among all cell lines, whereas RPMI8226 and AMO1 displayed significantly lower ARRS values. Consequently, we selected NCIH929 cells with high ARRS and RPMI8226, along with AMO1, which had a low ARRS, for further investigation. We assessed their responsiveness to drugs predicted to be tolerated in high-ARRS patients through oncoPredict analysis.

Subsequently, we exposed the three cell lines to a gradient of concentrations of seven anticancer drugs, including pictilisib, AZD5356, urosertib, nutlin-3a, selumetinib, foretinib, and dabrafenib, for a 72-h treatment period. The results indicated that, with the exception of AZD5356, NCIH929 exhibited the poorest response compared to the other two cell lines among the six drugs (Fig. 5F). IC50 values were calculated based on dose-response curves, and their standardized values were visualized in a heatmap, providing a clearer assessment of NCIH929’s relative resistance compared to the other two cell lines (Fig. 5G). Based on these findings, we have preliminarily confirmed *in vitro* that MM cell lines with a high ARRS are more tolerant to both protease inhibitors and inhibitors targeting the PI3K/Akt/mTOR signaling pathway.

### Role of KIF14 in cell proliferation, apoptosis and adhesion in MM

**Figure 6:**
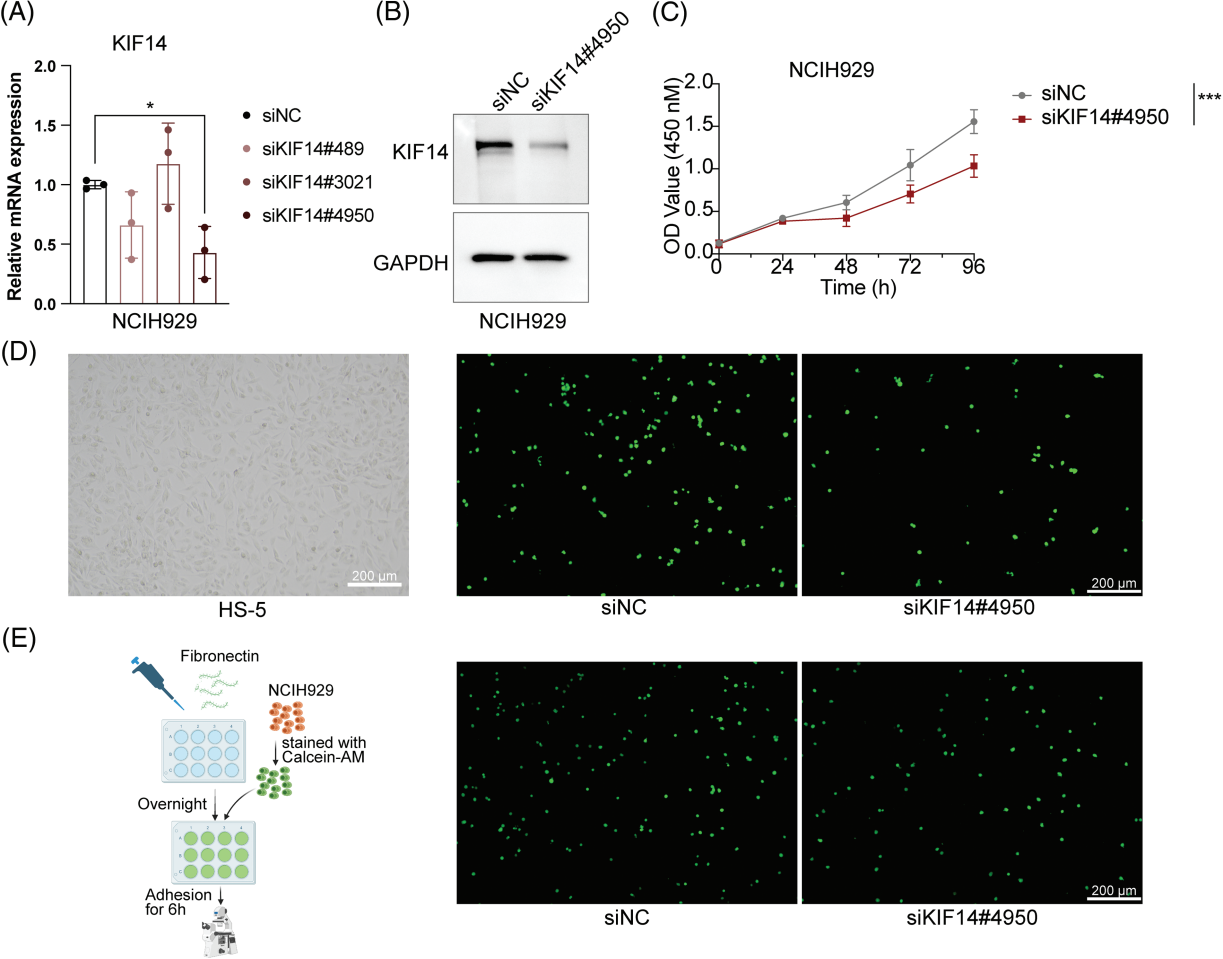
KIF14 knockdown suppresses proliferation and alters the adhesion of NCIH929 cells. (A) Validation of KIF14 knockdown in siKIF14#4950-transfected cells using qPCR analysis. (B) Representative western blot assessing KIF14 protein levels following transfection with siKIF14#4950 and siNC control in NCIH929 cells. (C) Cell proliferation curve displaying normalized OD450 values at 24-h intervals. (D) Images illustrating representative HS-5 monolayer morphotypes and siNC-or siKIF14#4050-transfected cells adhering to HS-5 cells. (E) Left flowchart depicting the adhesion assay procedure with fibronectin (FN), generated with BioRender (https://biorender.com). Right fluoromicrographs showing FN-adhered siNC-or siKIF14#4950-transfected NCIH929 cells. *p < 0.05, ***p < 0.001.

Based on our previous coexpression network and Lasso Cox regression analysis, we identified the KIF14 gene as playing a significant role in MM cell adhesion. To investigate its function, we utilized siRNA to silence KIF14 expression in NCIH929 cells. Among the three siRNA sequences targeting KIF14, only siKIF14#4950 effectively reduced both the gene and protein expression levels (Figs. 6A and 6B). Cell viability assays demonstrated that KIF14 knockdown led to a marked inhibition of NCIH929 cell proliferation (Fig. 6C). However, Annexin V-FITC and PI staining revealed no substantial increase in apoptosis or cell death (Suppl. Fig. S6). Previous studies have reported varying effects of KIF14 knockdown on apoptosis and proliferation inhibition, suggesting a context-dependent response based on gene silencing efficiency and cell cycle phase [44–46]. Our findings observed that KIF14 primarily inhibits MM cell proliferation but not apoptosis, consistent with its known role in cytoplasmic division and chromosome dynamics.

Moreover, research on solid tumors has shown that KIF14 knockdown can suppress tumor invasion and migration [44,47]. We observed similar effects in MM cells, where KIF14 knockdown significantly reduced adhesion to both bone marrow stromal cells (HS-5) and fibronectin-coated surfaces (Figs. 6D and 6E). Notably, KIF14, a molecular motor protein involved in intracellular transport, has been implicated in regulating cell adhesion by modulating the expression and membrane localization of cadherin 11 [47]. While studies on KIF14’s role in MM cell adhesion are limited, our results highlight its importance in this context. In summary, our study demonstrates that KIF14 knockdown inhibits cell proliferation and adhesion in NCIH929 cells, shedding light on its crucial role in MM pathology. These findings provide valuable insights into potential therapeutic strategies targeting KIF14 in MM.

## Discussion

Through bioinformatics analysis, our study identified 18 prognosis-related ARGs and successfully constructed an ARRS model using 12 of these genes, which independently predicts the prognosis of MM patients. Remarkably, the ARRS model outperforms conventional clinical parameters in predictive accuracy. In-depth analysis of the transcriptome data of patients in the high-and low-ARRS groups revealed that elevated ARRS may be associated with increased DNA damage repair capacity and activation of the P53 signaling pathway, leading to resistance to DNA-damaging drugs and inhibitors targeting the PI3K/AKT signaling pathway. Importantly, several of these candidate drugs were subsequently validated through *in vitro* cell experiments. Furthermore, our study predicts potential antitumor drugs that can reverse cell adhesion effects, providing new strategies to address CAM-DR in clinical settings. Additionally, our research provides pioneering evidence of KIF14’s pivotal role in suppressing MM proliferation and inhibiting adhesion, offering crucial insights for therapeutic interventions.

The progression of MM is heavily influenced by the bone marrow (BM) microenvironment. Research has demonstrated that the BM microenvironment is a critical factor in the development and progression of MM. MM cells interact with various adhesion molecules and the ECM within the BM, supporting mechanisms related to MM pathogenesis, drug resistance, and cell migration [48]. During the clonal evolution of MM, it is initiated by mutations associated with T-cell-dependent B-cell activation. Subsequently, MM cells home to the BM through the interaction of the MM receptor CXCR4 with the chemokine SDF1α. Within the BM microenvironment, MM cells interact with various components, including BM stromal cells (BMSCs) and ECM proteins such as fibronectin, collagen, osteopontin, hyaluronan, and laminin, which facilitate MM survival, proliferation, migration, and drug resistance through the MEK/MAPK, JAK/STAT, and PI3K/Akt pathways [49,50]. Furthermore, the interaction between MM cells and cancer-associated fibroblasts (CAFs) via adhesion molecules such as CXCL12/CXCR4 and integrins is essential for promoting CAFs’ tumor-supporting functions. Recent studies have identified cell adhesion as a significant marker of extramedullary myeloma [51].

Our research highlights the importance of cell adhesion in the survival prognosis of MM. Elevated cell adhesion effects in our study influenced the activation of the DNA replication process in MM. Dysregulated cell adhesion may lead to altered cell cycle regulation, genomic instability, and increased DNA damage, as reported in previous research [52]. CAM-DR is particularly relevant in hematologic malignancies, such as MM. Adhesion molecules, such as VLA-4, play a crucial role in CAM-DR. The interaction between VLA-4 and vascular cell adhesion molecule-1 (VCAM-1) mediates binding between multiple myeloma cells and BMSCs, contributing to the survival of multiple myeloma cells via the activation of the PI3K/AKT pathway and CAM-DR [53,54]. In line with the above, our drug sensitivity prediction results revealed that high-ARRS MM exhibits significantly poor sensitivity to PI3K/AKT pathway-targeted drugs, as well as lower sensitivity to DNA-damaging agents compared to the low-ARRS group. Therefore, we recommend analyzing and testing the cell adhesive effects in patients with MM in the future. For MM patients with high cell adhesion, it is advisable to avoid using DNA damaging drugs or PI3K/AKT pathway inhibitors whenever possible. Furthermore, we look forward to conducting further animal and clinical experiments to validate the candidate drugs useful in high-ARRS MM, which will provide CAM-DR patients with more treatment options.

MM exhibits significant heterogeneity in both biological and clinical contexts, and currently lacks a universally accepted risk stratification tool. The widely utilized ISS is primarily based on levels of albumin and β2-microglobulin. In 2016, the Revised International Staging System (R-ISS) was introduced, combining tumor burden (ISS) with disease biology. However, cases characterized by the t(4;14) translocation and abnormal gain(1q) experience a notable decrease in survival rates. The impact of advanced ISS staging, high-risk IgH translocation, and 1q gain on five-year survival rates in diagnosed patients is no longer statistically significant [55]. These findings suggest that there are still numerous unknown prognostic factors influencing the ultimate outcome of the disease. Overreliance on cytogenetic abnormalities for prognostic assessment may pose certain challenges, indicating a need for improvement in the current prognostic evaluation systems. In our study, we constructed an ARRS model that independently predicts the prognosis of MM patients. Furthermore, we integrated the ARRS, ISS, and other clinical parameters into a nomogram, demonstrating a high level of accuracy in predicting survival outcomes for MM patients. Therefore, the prognostic stratification tool we provide may help address the shortcomings of current prognostic analysis and evaluation systems, offering a more in-depth characterization of disease biology and prognosis.

A previous study conducted a bioinformatics-based analysis to identify cell adhesion genes closely associated with the diagnosis and prognosis of MM [56]. They screened MM-related differentially expressed genes using the GSE6477 dataset, comprising 147 MM patients and 15 healthy individuals. Functional enrichment analysis revealed a pivotal role of cell adhesion mechanisms in MM pathogenesis. Through PPI network analysis, 12 adhesive genes were identified as potential hub genes, confirming their diagnostic value for MM. Notably, ITGA9 and LAMB1 showed significant associations with disease-specific survival in MM. However, this study lacked validation across multiple databases and primarily relied on molecular interactions rather than direct disease associations. Additionally, no specific diagnostic or prognostic tools related to cell adhesion were constructed. In contrast, our study focused on elucidating the role of cell adhesion effects in MM prognosis. We employed a Lasso regression model to construct the cell adhesion-related risk model, known for variable selection and regularization to enhance prediction accuracy and model interpretability [57]. This approach provides a broader context by considering the coordinated actions of multiple genes within a functional module, offering deeper insights into the underlying biology and potential therapeutic targets. Consequently, our research encompassed a wider array of datasets and examined the function of cell adhesion with a more stringent selection process and comprehensive perspective. However, our study does have limitations. It is based on publicly available data from online databases, and there is a lack of validation using large-sample data from local clinical centers.

Our study is the first to comprehensively analyze the role of cell adhesion-related genes in the prognosis of MM using bioinformatics approaches. This finding revealed that high cell adhesion effects may serve as adverse prognostic factors in MM. Furthermore, our investigation identified potentially ineffective drugs in patients with high adhesion effects and proposed candidate strategies for MM treatment. Thus, our findings furnish a theoretical basis for stratified and personalized treatment in MM.

## Supplementary Materials

FIGURE S1 Univariate Cox regression analysis of 18 overlapping prognostic adhesion-related genes. Forest plots showing the univariate Cox regression results in GSE2658, GSE9782 and GSE136337.

FIGURE S2 Distribution of adhesion-related risk scores in diverse patients. (A) Distribution of adhesion-related risk scores in multiple myeloma patients in the CoMMpass, GSE136337, GSE9782 and GSE2658 datasets. (B) Scatter plots visualizing the distribution of patients with diverse survival statuses.

FIGURE S3 Construction of a prognostic nomogram based on the adhesion-related risk score and clinical parameters. (A) Nomogram combining the risk score with significant clinicopathologic features to predict the overall survival of MM patients in the CoMMpass and GSE136337 datasets. (B) Calibration curves of the nomogram model in the CoMMpass and GSE136337 datasets.

FIGURE S4 Role of ARRS model genes and activation of adhesion-related pathways in diverse ARRS MM patients. (A) Molecular function analysis of the 12 genes included in the ARRS model using Funrich. (B) Gene set enrichment analysis (GSEA) showing the enrichment of cell adhesion-related pathways in the high-ARRS group compared to the low-ARRS group. ES, enrichment score. NES, normalized enrichment score.

FIGURE S5 Functional enrichment analysis based on the prognostic adhesion-related risk model in the GSE136337 dataset. (A) Heatmap visualizing the enrichment scores of gene set variation analysis (GSVA) in the GSE136337 cohort. (B) Bubble plot showing the significantly enriched KEGG pathways in the high-risk group compared to the low-risk group identified by gene set variation analysis (GSVA) in the GSE136337 cohort.

FIGURE S6 Representative Flow Cytometry Profiles. NCIH929 cells were transfected with either siNC or siKIF14#4950 for 48 hours. Subsequently, cells were stained with Annexin V-FITC and PI to distinguish apoptotic (Q2) and necrotic (Q3) cell populations.

## Data Availability

The datasets utilized and/or analyzed during the present study will be made accessible by the corresponding author upon reasonable request.

## Supplementary Materials

The supplementary material is available online at https://doi.org/10.32604/or.2023.043647.
