# Supplementary figures and images for: Development of a cell adhesion-based prognostic model for multiple myeloma: Insights into chemotherapy response and potential reversal of adhesion effects
Source: Oncol Res. 2024 Mar 20;32(4):753–68. doi: 10.32604/or.2023.043647 (PMC10972724; doi:10.32604/or.2023.043647)

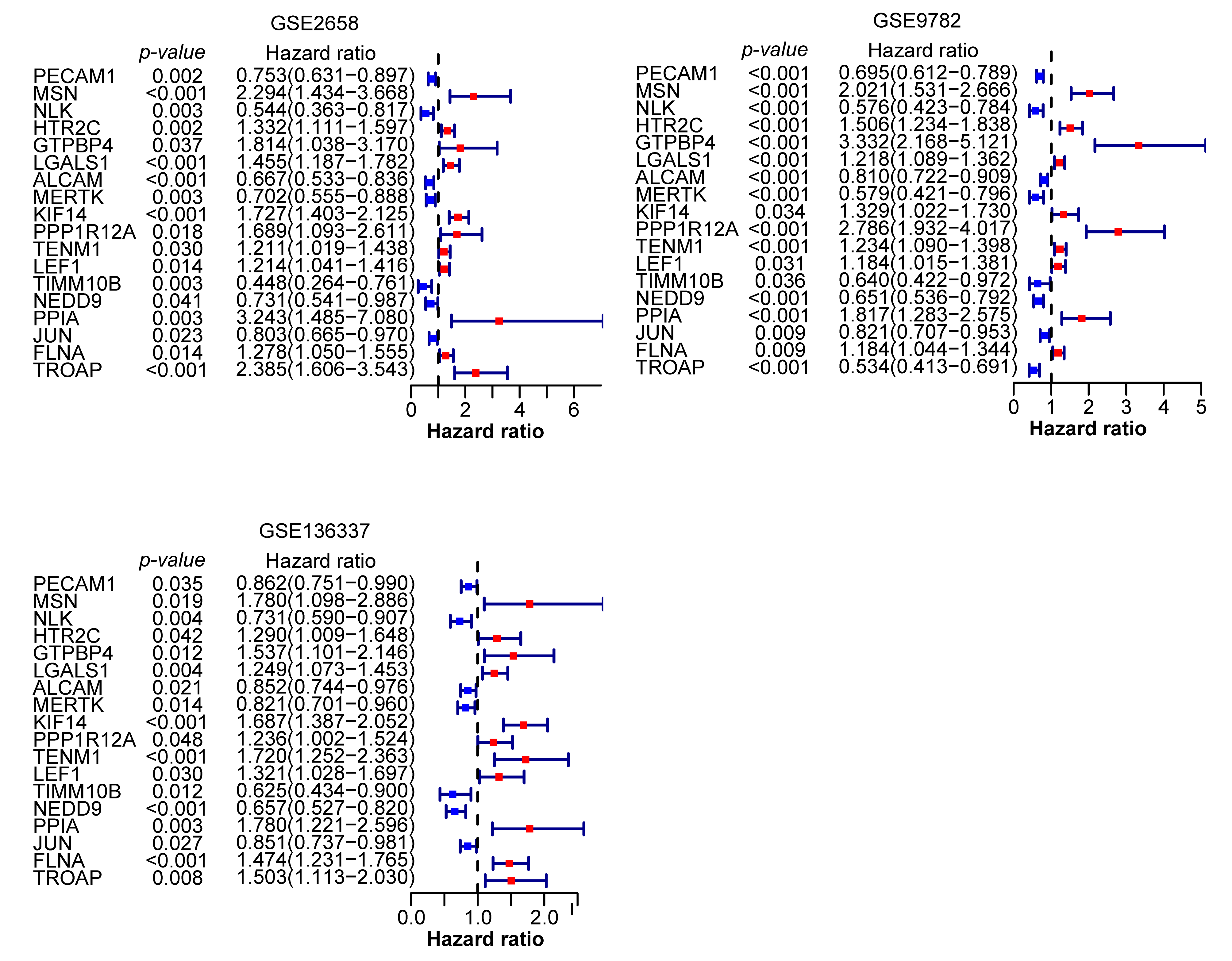

Supplement: FIGURE S1 [file OncolRes-32-43647-s001.tif]

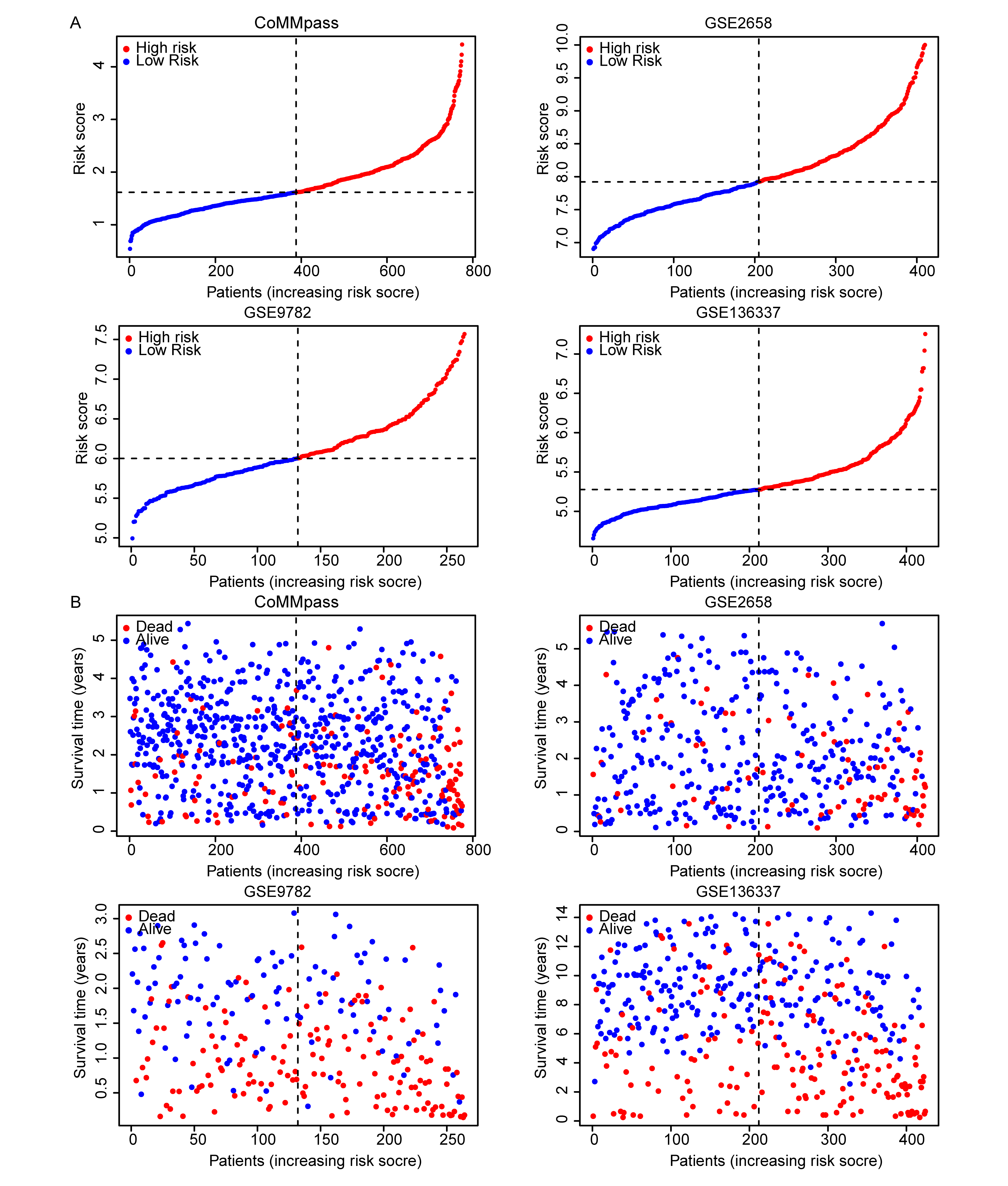

Supplement: FIGURE S2 [file OncolRes-32-43647-s002.tif]

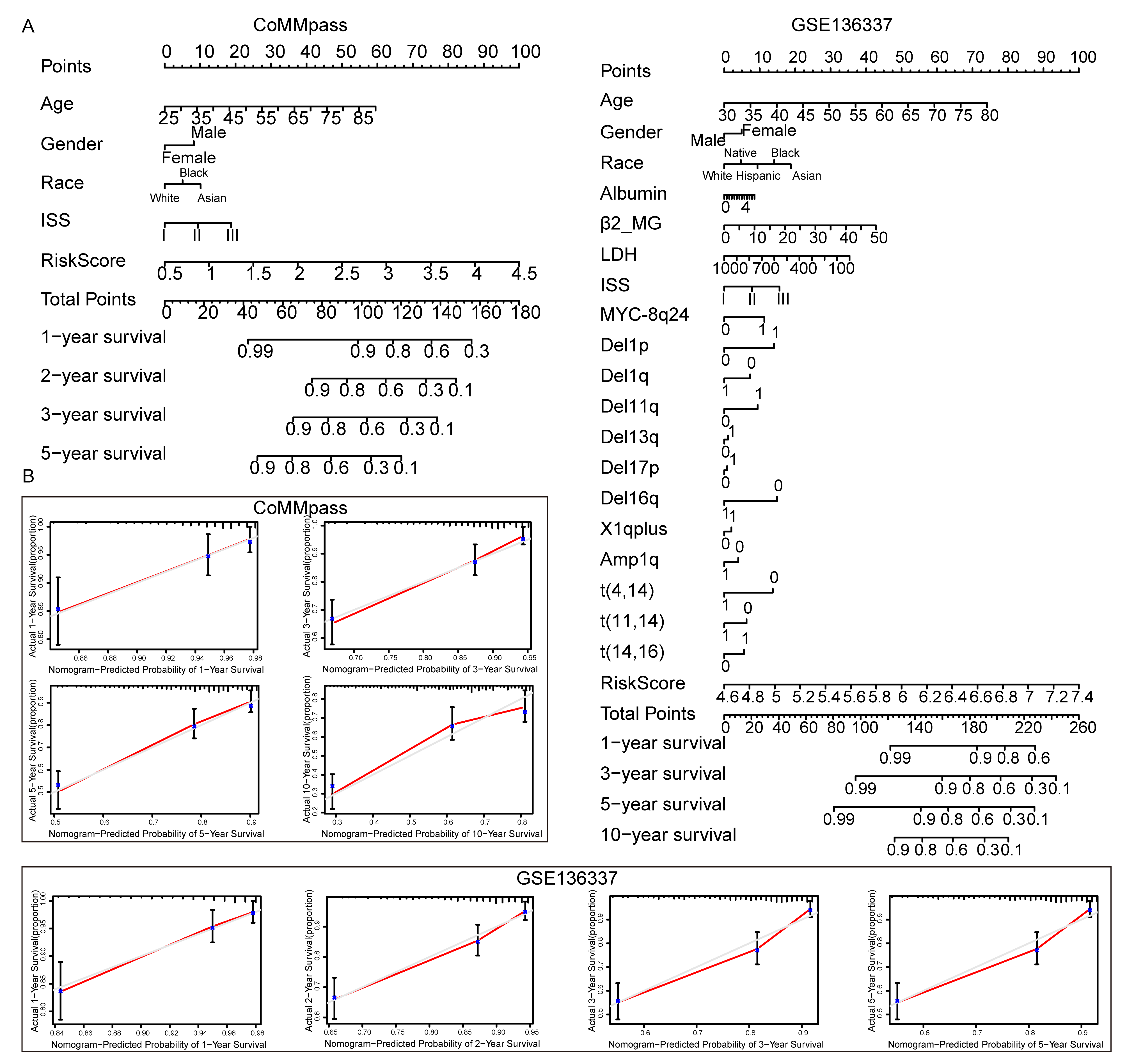

Supplement: FIGURE S3 [file OncolRes-32-43647-s003.tif]

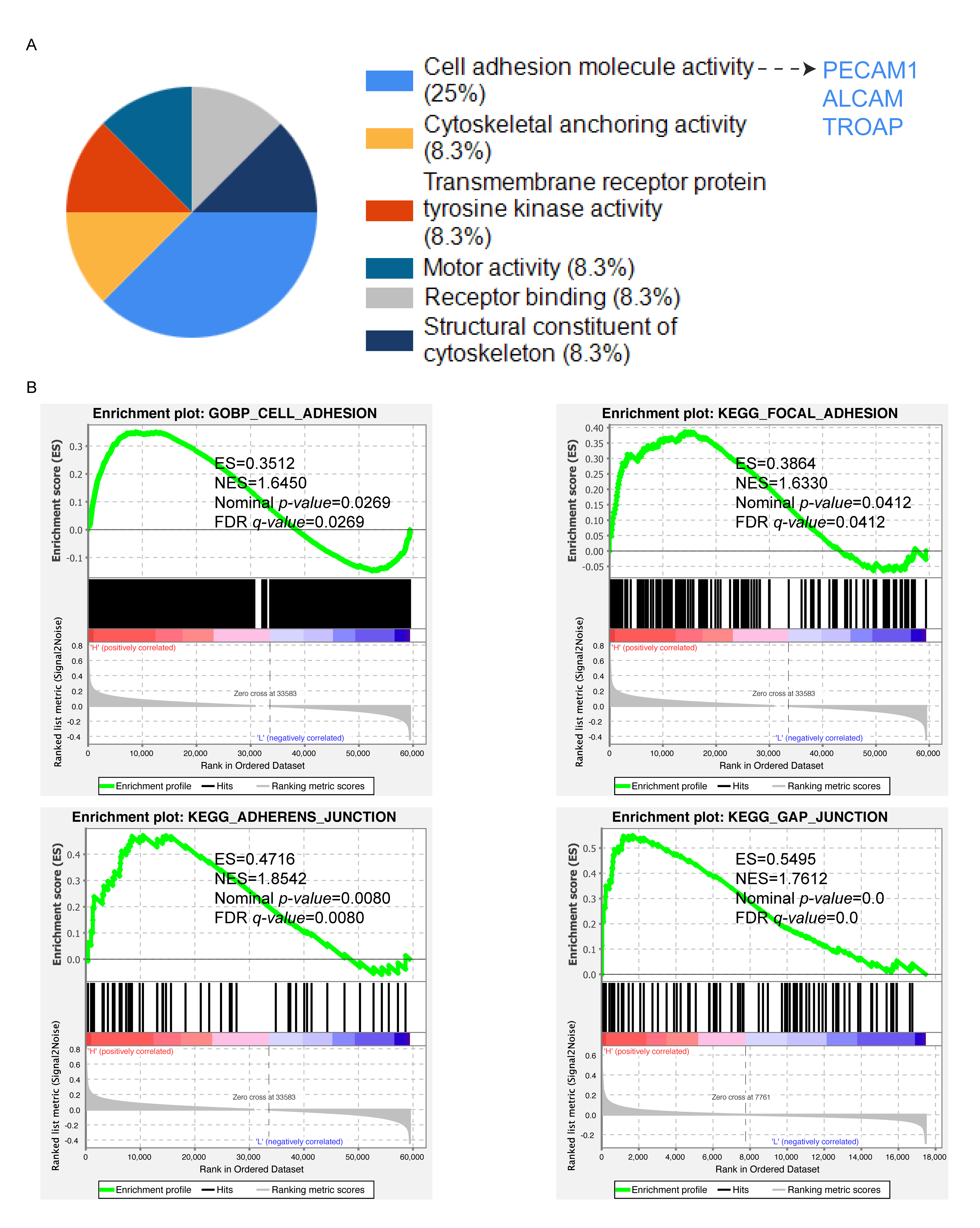

Supplement: FIGURE S4 [file OncolRes-32-43647-s004.tif]

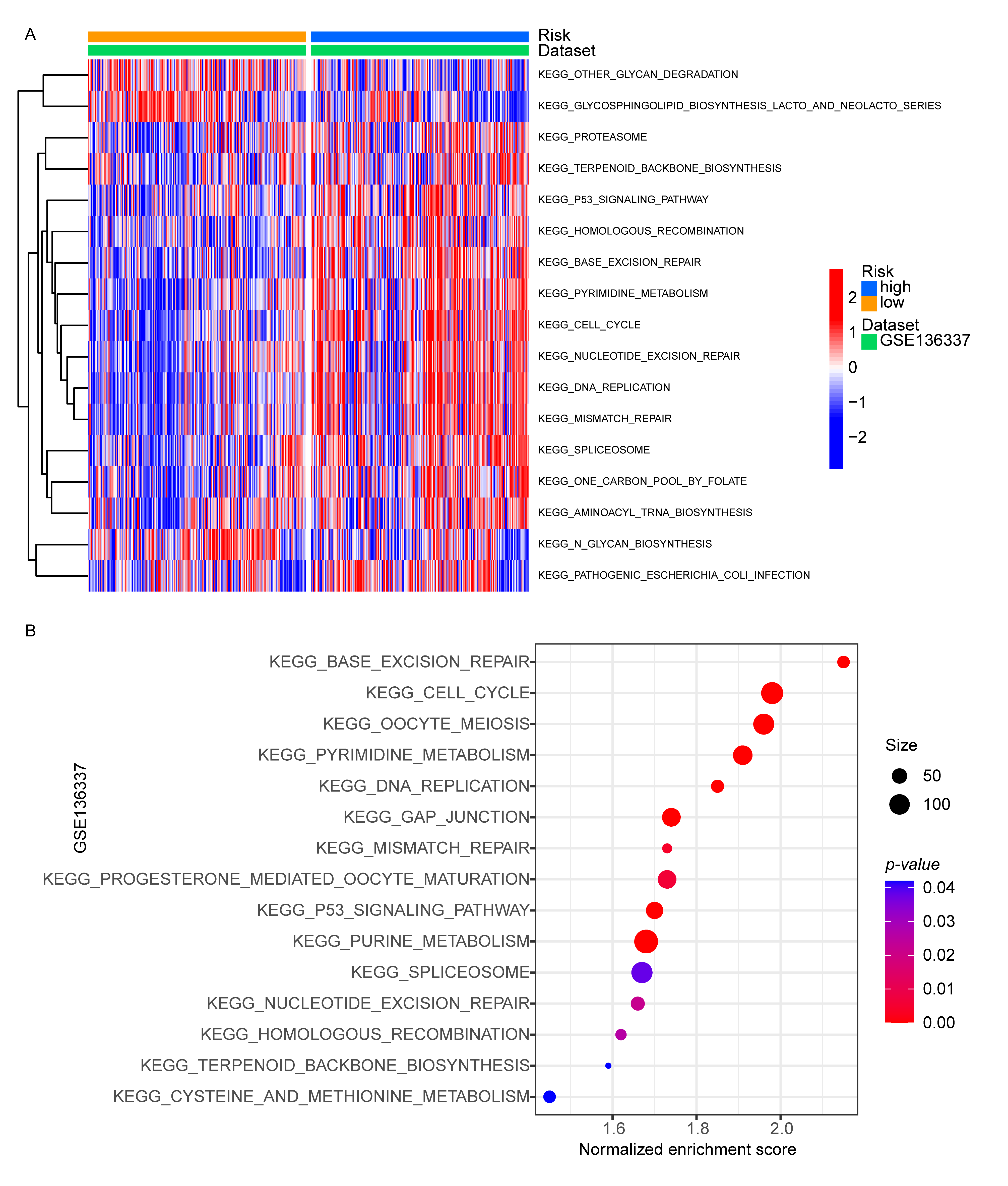

Supplement: FIGURE S5 [file OncolRes-32-43647-s005.tif]

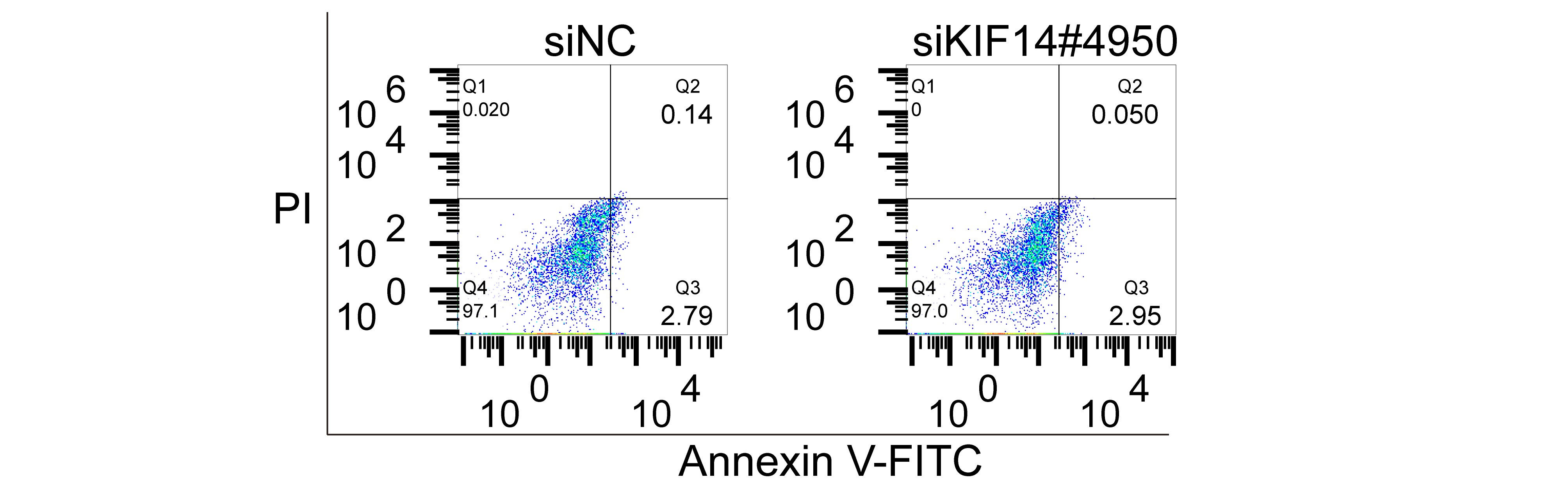

Supplement: FIGURE S6 [file OncolRes-32-43647-s006.tif]
